# 

**Published:** 1851-03

**Authors:** 


WHOLE SERIES—VOL. VII, NO. VI.

VOL. III.] MARCH, 1851. [No. 6.

### THE NORTH-WESTERN MEDICAL AND SURGICAL JOURNAL.

PUBLISHED EVERY OTHER MONTH.

AT TWO DOLLARS PER ANNUM IN ADVANCE.

EDITED BY

JOHN EVANS, M. D.,

PROFESSOR OF OBSTETRICS ETC., IN RUSH MEDICAL COLLEGE,

AND

EDWIN G. MEEK, A. M., M. D.

CHICAGO AND INDIANAPOLIS:

PRINTED BY JAS. J. LANGDON,

161 Lake street, Chicago.

1851.

ENLARGED SERIES—VOL. V, NO. VI.

